# Site-specific differences in the association between plantar tactile perception and mobility function in older adults

**DOI:** 10.3389/fnagi.2014.00068

**Published:** 2014-04-11

**Authors:** Yenisel Cruz-Almeida, Mieniecia L. Black, Evangelos A. Christou, David J. Clark

**Affiliations:** ^1^Pain Research and Intervention Center of Excellence, University of FloridaGainesville, FL, USA; ^2^Department of Aging and Geriatric Research, University of FloridaGainesville, FL, USA; ^3^Department of Applied Physiology and Kinesiology, University of FloridaGainesville, FL, USA; ^4^Brain Rehabilitation Research Center, Malcom Randall VA Medical CenterGainesville, FL, USA

**Keywords:** somatosensation, aging, mobility, walking, balance

## Abstract

**Introduction:** Impaired somatosensation is common in older adults and contributes to age-related loss of mobility function. However, little is known about whether somatosensation at different sites on the plantar surface of the foot are differentially related to mobility function. Such a finding may have important implications for clinical care of older adults and other at-risk populations, such as for optimizing interventions (e.g., footwear for augmenting somatosensory feedback) and for improving the efficiency of clinical assessment.

**Materials and Methods:** Tactile perception was evaluated with a 10 g monofilament at four sites on the plantar surface of each foot: great toe (GT), first metatarsal head (MT1), heel (H) and fifth metatarsal head (MT5). Mobility function was assessed with the Berg Balance Scale and walking speed.

**Results:** Sixty-one older adults participated. Tactile perception was significantly positively associated with Berg Balance Score (adjusted *r* = 0.30 − 0.75; *p* = 0.03 − < 0.001), with the strongest association found at the site of the MT1. Only at this site was tactile perception found to be significantly associated with usual walking speed (adjusted *r* = 0.51; *p* < 0.001) and maximal walking speed (adjusted *r* = 0.38, *p* = 0.004). Clinically mild somatosensory impairment at MT1, but not at other sites, was found to yield substantial deficits in both Berg Balance Score and walking speed.

**Discussion:** The present findings indicate that tactile perception at MT1 is more closely linked to mobility function than is tactile perception at GT, MT5 or H. These findings warrant further research to examine whether interventions (e.g., textured insoles) and assessments that preferentially or exclusively focus on the site of MT1 may be more effective for optimizing clinical care.

## Introduction

Impaired somatosensation is common in older adults (Mold et al., 2004; Shaffer and Harrison, 2007). A large study of peripheral nerve function in older adults found bilateral somatosensory deficits in approximately 26% of individuals 65–74 years of age, 36% of individuals 75–84 and 54% of individuals ≥85 (Mold et al., 2004). This age-related impairment of somatosensation has important functional implications for older adults, as it has been linked to deficits in balance and walking ability (Resnick et al., 2000; Mold et al., 2004; Deshpande et al., 2008; Buchman et al., 2009). A particular concern with somatosensory impairment is increased risk of injurious falls (Sorock and Labiner, 1992; Richardson and Hurvitz, 1995), which are a major contributor to disability and death in older adults (Soriano et al., 2007). The issue is compounded by the fact that many older adults are unaware that they have peripheral neurological impairment (Mold et al., 2004), and are therefore unlikely to seek preemptive intervention.

Given the importance of somatosensory function to mobility in older adults, there is a clear need for research that can contribute to enhanced clinical assessment and intervention of somatosensory deficits. A potentially important question is whether mobility outcomes are differentially affected by the site of somatosensory deficits. For example, the presence of site-specific differences in the relationship with balance or walking function could have important implications for intervening with sensory augmentation footwear. Sensory augmentation footwear involves placing specialized insoles in the shoes (i.e., textured or vibrating insoles), and has shown promise for enhancing mobility function in older adults (Priplata et al., 2003; Palluel et al., 2008, 2009; Qiu et al., 2012; Stephen et al., 2012; Hatton et al., 2013). However, there are also studies which report no benefit or possibly even deleterious effects of somatosensory augmentation (Hatton et al., 2009, 2012; Hartmann et al., 2010). Knowledge of site-specific associations with mobility function may be helpful for explaining such discrepancies and for guiding future development of footwear. This knowledge may also be valuable for refining clinical assessment protocols so that somatosensory screening can be conducted in a time and cost-effective manner. Accordingly, the objective of the present study is to investigate the extent to which tactile perception at four different sites on the plantar surface of the foot may be differentially associated with mobility function, including tests of balance and walking speed.

## Materials and methods

### Participants

Older adult volunteers were recruited by newspaper advertisement and mass mailing to a research recruitment database. The database consisted of a heterogeneous mix of older adults who had previously indicated willingness to volunteer for research studies. Screening of inclusion/exclusion criteria was conducted by telephone. Inclusion criteria for this study were: (1) age between 65 and 85 years and (2) agreement with the statement “You find it physically tiring to walk a quarter mile, or climb two flights of stairs, or perform household chores (at least one of these should be true)”. Exclusion criteria included use of an assistive device for walking (cane, crutch, walker, brace, etc.); lower extremity pain while walking; involuntary weight gain or loss exceeding 10 pounds within the past 6 months; myocardial infarction or symptomatic cardiovascular disease in the past year; bone fracture in the past year; injury or illness to the central nervous system; uncontrolled hypertension exceeding 160 systolic and/or 95 diastolic; or terminal illness. Volunteers who met these criteria were invited to our research center to participate in the assessments described below. During this visit, we also acquired demographic and health-related information (e.g., medication use, weight and height) and administered the Mini Mental State Examination to assess cognitive status. In order to determine if volunteers were aware of the presence of a clinically significant neuropathy, they were asked to reply “Yes” or “No” to the question: “Do you regularly experience either numbness or tingling in your feet (when sitting or walking)?” All study procedures were approved by the University of Florida Institutional Review Board. All individuals participating in on-site assessments provided their written informed consent.

### Assessment of cutaneous tactile perception

**Figure 1:**
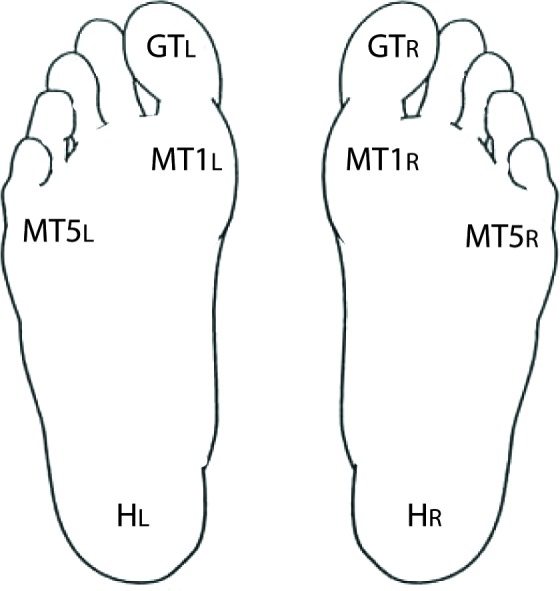
**Testing sites for tactile perception**. Using the figure shown, participants were instructed to report which site was touched by the monofilament. A total of 24 trials were conducted, including 16 with a tactile stimulus and 8 sham trials with no stimulus.

Cutaneous tactile perception was assessed at four sites on the plantar surface of each foot using a Semmes–Weinstein 5.07 (10 g) monofilament. The sites tested were the great toe (GT), first metatarsal head (MT1), fifth metatarsal head (MT5) and heel (H). Participants laid flat on their back on an examination table. They were provided with an illustration of a set of “footprints” (Figure 1), with each testing site labeled with a code that specified side and testing site. When prompted by the examiner, the participants were instructed to indicate whether they felt a touch and, if so, at what site. The examiner performed 24 trials (three trials at each of the four sites for both feet) in random order. At each site, one of the three trials was a “sham” in which the examiner did not actually touch the participant but still asked for a response. One point was awarded for each correct response (including a “no” response for a sham trial) for a total cutaneous tactile score of up to 24 points (12 points per foot). For ease of interpretation, the number of correct responses was converted to a percent value.

### Functional measures

Balance was assessed with the Berg Balance Scale (Berg et al., 1989), which is a 14-item performance assessment of balance related tasks. Each task is scored on an ordinal scale from 0 (unable) to 4 (independent). The sum of all scores is used as the final outcome. Both walking tests (usual and maximal speed) were timed with a stopwatch. In order to ensure that we recorded steady state walking speed during the 10 m fast walk, the beginning and end of the course included 5 m acceleration/deceleration zones such that the total distance walked was 20 m. The watch was started and stopped when the individual’s torso passed over the lines marking the start and end, respectively, of the 10 m course. Three fast walking trials were conducted, and the average was used for subsequent analysis. Only one trial was conducted for 400 m usual walking speed.

### Statistics

Values are reported as mean ± standard deviation unless otherwise stated. The data were checked for normality and homoscedasticity. Consistency of results for left and right sides (i.e., inter-item reliability) was assessed by examining Cronbach’s α coefficients. For the primary analysis, associations between continuous variables were quantified by Pearson’s moment correlations, and partial correlations accounted for age, sex, body mass index, MMSE score and number of medications. Regression models were used to compare the strength of associations between different somatosensory sites and mobility tests, after adjusting for confounding variables. Statistical analysis was conducted using SPSS 21, and significance level was set to α < 0.05. For the secondary analysis, two tailed *t*-tests were used to compare subgroups of participants. The False Discovery Rate Procedure was used to correct for multiple comparisons (Curran-Everett, 2000).

## Results

### Participant characteristics

A total of 198 individuals underwent screening by telephone. Of these, 72 passed the phone screen and were invited to our research center for on-site screening and assessment. Ultimately, 61 (38 females and 23 males) were found to meet all criteria and were able to complete all necessary assessments. The mean age was 74.5 ± 6.6 years. The mean body mass index was 28.0 ± 3.6 (range 16.2–34.5) and the mean score on the Mini-Mental State Exam was 27.5 ± 1.7 (range 24–30). Participants used on average 3 prescription medications (range 0–13 medications).

### Somatosensory function

Tactile perception data were examined for the statistical assumptions of normality, independence and homoscedasticity. The data were found to be significantly non-normally distributed (skewness statistic = −1.961 ± 0.31, *p* < 0.01), and were transformed with a square root transformation prior to conducting regression analysis. Tactile perception for the left and right sides (including data from all four testing sites) were significantly correlated (*r* = 0.73, *p* < 0.001), with high inter-item reliability (Cronbach’s α = 0.85). Therefore, the left and right sides were averaged by testing site to yield four tactile perception scores, which were used for subsequent analysis. The group mean scores for each testing site were: GT: 86.1 ± 22.2%, MT1: 89.0 ± 21.1%, H: 87.1 ± 21.0% and MT5: 90.6 ± 19.9%. Scores from the four sites were significantly positively associated with one another, with correlations ranging from 0.48 to 0.62 (*p* < 0.001). Seventeen participants (out of 61) had at least one false positive response to the sham trials, with the overall proportion of false positive responses being about 6%.

Approximately 37% of participants self-reported regularly experiencing tingling and/or numbness in their feet (i.e., abnormal somatosensation). Tactile perception at MT1 was significantly worse in those reporting abnormal somatosensation compared to those who did not report this deficit (81.8% vs. 95.2%, *p* = 0.005). Similar non-significant trends were also observed at MT5 (*p* = 0.05) and GT (*p* = 0.11).

### Relationship between somatosensation and mobility function

Tactile perception at each site was significantly associated with performance on the Berg Balance Scale. These findings were maintained after adjusting for age, sex, body mass index, MMSE score and medication use (Table 1). Tactile perception at MT1 was the only site found to be significantly associated with usual and maximal walking speed. The strength of the association between Berg Balance score and tactile perception at MT1 was significantly stronger than the associations between Berg Balance score and tactile perception at each other site (adjusted *p* < 0.05). The results also show that the strength of the association between MT1 and Berg Balance score was stronger than the association between MT1 and maximal speed (adjusted *p* < 0.05). The same trend was evident for the relationship between MT1 and Berg Balance score relative to that of MT1 and usual walking speed (adjusted *p* = 0.07).

**Table 1:** **Associations between somatosensory scores and mobility function**.

	**GT**	**MT1**	**H**	**MT5**
Adjusted r*				
Berg Balance Score	0.41, *p* < 0.002	0.75, *p* < 0.001	0.39, *p* = 0.003	0.30, *p* = 0.03
400m Usual (m/s)	0.17, *p* = 0.22	0.51, *p* < 0.001	0.17, *p* = 0.21	0.07, *p* = 0.59
10m Maximal (m/s)	0.11, *p* = 0.44	0.38, *p* = 0.004	0.00, *p* = 0.98	−0.15, *p* = 0.25

* adjusted for age, sex, body mass index, MMSE score and number of medications

Abbreviations:
GT (great toe); MTI (first metatarsal head); H (heel); MT5 (fifth metatarsal head)

A secondary analysis was conducted to examine the extent to which the occurrence of clinically mild somatosensory impairment at each plantar testing site may affect mobility function. For each testing site, we compared subgroups of older adults with no clinically detectable impairment (i.e., no incorrect responses at the plantar site) to those with clinically mild impairment (i.e., only one incorrect response at the plantar site). Results of this analysis are shown in Table 2. Compared to participants with no impairment at MT1, those with mild impairment at MT1 had lower scores for the Berg Balance Scale (53.0 ± 3.1 vs. 50.3 ± 3.0, *p* = 0.006), usual walking speed (1.12 ± 0.12 vs. 0.97 ± 0.12 m/s, *p* = 0.007), and a trend for lower maximal walking speed (1.61 ± 0.23 vs. 1.45 ± 0.37 m/s, *p* = 0.073). For each other site (GT, H, MT5) there were no statistically significant differences for any mobility test between the subgroup with no impairment vs. the subgroup with mild impairment (all *p* > 0.17).

**Table 2:** **Mobility function for subgroups without somatosensory impairment versus with mild somatosensory impairment**.

	**GT**		**MT1**		**H**		**MT5**	
**Not Impaired (n = 40)**	**Mildly Impaired (n = 10)**	**Not Impaired (n = 41)**	**Mildly Impaired (n = 11)**	**Not Impaired (n = 37)**	**Mildly Impaired (n = 14)**	**Not Impaired (n = 44)**	**Mildly mpaired (n = 10)**
Berg Balance Score	52.0 ± 3.6	52.3 ± 3.5	***53.0 ± 3.1***	50.3 ± 3.0*	52.8 ± 3.4	50.1 ± 5.5	52.1 ± 3.5	50.9 ± 6.0
Usual Speed (m/s)	1.09 ± 0.13	1.04 ± 0.14	***1.12 ± 0.12***	0.97 ± 0.12*	1.07 ± 0.13	1.05 ± 0.19	1.07 ± 0.12	1.09 ± 0.20
Maximal Speed (m/s)	1.59 ± 0.29	1.5 ± 0.16	1.61 ± 0.23	1.45 ± 0.37	1.55 ± 0.26	1.58 ± 0.37	1.53 ± 0.22	1.62 ± 0.44

* indicates adjusted * p < 0.05.

Abbreviations:
GT (great toe); MTI (first metatarsal head); H (heel); MT5 (fifth metatarsal head)

## Discussion

The primary objective of this study was to investigate the extent to which tactile perception at four different sites on the plantar surface of the foot may be differentially related to mobility function in older adults. The results indicate that tactile perception at MT1 is more closely linked to mobility function than is tactile perception at GT, H or MT5. The strength of the association between Berg Balance score and tactile perception at MT1 was significantly stronger than the associations between Berg Balance score and tactile perception at each other site. Furthermore, MT1 was the only site that was significantly associated with usual and maximal walking speed. What accounts for the finding that tactile perception at MT1 is especially highly linked to mobility function? It might be explained in part by the prior finding that somatosensory thresholds are lower (i.e., more sensitive) in the ball of the foot, which includes MT1, compared to the heel and toe regions (Inglis et al., 2002). This could be due to the relatively high density of slow and fast adapting cutaneous receptors at MT1 (Kennedy and Inglis, 2002; Fallon et al., 2005). These anatomical characteristics may be an evolutionary adaptation in response to the important role of the forefoot in control of walking. The forefoot, including MT1, is highly involved in the crucial biomechanical task of forward propulsion during gait (Melai et al., 2013). In this role, the forefoot bears high levels of pressure that likely provides important neural input that is relevant to controlling speed and directional steering during walking.

Even clinically mild somatosensory impairment at MT1 appears to have a marked influence on mobility function. This was evidenced by our subgroup analyses of individuals with clinically mild somatosensory impairment vs. those with no clinically detectable somatosensory impairment. For tactile perception at MT1, Berg Balance Scale score was 2.7 points lower for the subgroup with mild impairment vs. the subgroup with no impairment (50.3 vs. 53.0 points). This difference approaches the value of 3.3 points that has been reported as a meaningful change for independently ambulating older adults (Donoghue and Stokes, 2009). Furthermore, the score of 53 in the subgroup with no impairment remains above the threshold of 51 that is indicative of a higher fall risk in elders who have a prior history of falling (Shumway-Cook et al., 1997). Equally alarming is the group difference of 0.15 m/s for usual walking speed, which well exceeds the threshold of 0.10 m/s that is considered a clinically meaningful change in older adults (Perera et al., 2006). The importance of MT1 relative to other plantar sites is further confirmed by the absence of significant differences in mobility function between subgroups at the other plantar sites.

Another interesting finding from this study is the distinct difference in the extent to which tactile perception is linked to Berg Balance score vs. walking speed. At sites GT, MT5 and H, there was a significant association between tactile perception and Berg Balance score, but not between tactile perception and walking speed. At site MT1, the strength of the association between tactile perception and Berg Balance score was significantly stronger than the association with maximal walking speed (*p* < 0.05) and there was a trend for a stronger association compared to usual walking speed (*p* = 0.07). The finding that somatosensation is more closely linked to balance is consistent with prior reports (Mold et al., 2004; Deshpande et al., 2008; Zhang and Li, 2013), although the reason remains unclear. It may be that processing of movement control for balance is less “automatic” than processing for walking, and relies more heavily on utilization of peripheral inputs to provide real-time information (Brandt et al., 1999; Jahn et al., 2004; Zwergal et al., 2012; Lau et al., 2014), such as for integration with the visual and vestibular systems. Another possible explanation is that there is an age-related increase in controlled processing of balance (Boisgontier et al., 2013) that is less pronounced for gait. It may also be that somatosensory information is more likely to be deficient during balance tasks. This is because activation thresholds of peripheral receptors are more likely to be reached during walking due to higher pressure and impact forces compared to balance tasks (Jonely et al., 2011).

Overall, the results of this study strongly agree with prior work demonstrating that impaired somatosensation is detrimental to mobility function (Resnick et al., 2000; Mold et al., 2004; Deshpande et al., 2008; Buchman et al., 2009). We have expanded on this knowledge by demonstrating site-specific differences in the association between tactile perception and mobility function. There are some methodological considerations that should be noted when interpreting the results of this study. We assessed somatosensation of the plantar surface of the feet because they are the primary interface between ground and body, and because peripheral age-related neural impairments are most profound distally (Shaffer and Harrison, 2007). However, the relationship between somatosensation and mobility may also be affected by other types of somatosensory information (e.g., muscle spindles and joint receptors) from other lower extremity regions. We used a clinical assessment of somatosensation (monofilament) as opposed to more rigorous assessments (e.g., sensory thresholds). Accordingly, our data should be interpreted in the context of clinically detectable levels of impairment.

The findings from this research may have important implications for clinical care of older adults, such as in the design of footwear that is used to augment somatosensation. Our finding that mobility function is most closely associated with somatosensory function at MT1 warrants further research to examine whether insoles that preferentially or exclusively augment somatosensation at MT1 provide an advantage for enhancing functional outcomes. The potential importance of this topic is supported by evidence showing that the site of somatosensory stimulation on the plantar surface of the foot can yield different postural responses (Kavounoudias et al., 1998, 2001). Our findings also have implications for clinical assessment. An ideal assessment of somatosensory function would involve testing at multiple sites with multiple modalities (tactile, vibratory, proprioception, etc.). However, this rigorous level of assessment may be excessively time consuming and costly for use in regular screenings. Single-site assessment is more feasible and may encourage more widespread use of somatosensory screening in older adults. Although more research is warranted, our data suggest that assessment at MT1 may prove to be the most relevant site in the context of gauging risk for adverse mobility outcomes.

## Author contributions

This study was designed by David J. Clark and Evangelos A. Christou. Data collection was conducted by Mieniecia L. Black and David J. Clark. Data analysis was conducted by Yenisel Cruz-Almeida and David J. Clark. The content of the manuscript was prepared by Yenisel Cruz-Almeida, Mieniecia L. Black, Evangelos A. Christou and David J. Clark.

## Conflict of interest statement

The authors declare that the research was conducted in the absence of any commercial or financial relationships that could be construed as a potential conflict of interest.
